# Developing digital interventions for people living with serious mental illness: perspectives from three mHealth studies

**DOI:** 10.1136/eb-2017-102765

**Published:** 2017-10-12

**Authors:** Bruno Biagianti, Diego Hidalgo-Mazzei, Nicholas Meyer

**Affiliations:** 1 Department of Psychiatry, University of California at San Francisco, San Francisco, California, USA; 2 Department of Health Sciences, University of Milan, Milan, Italy; 3 Research and Development, Posit Science Inc., San Francisco, California, USA; 4 Department of Psychiatry and Psychology, Bipolar Disorders Program, Institute of Neuroscience, Hospital Clinic, University of Barcelona, IDIBAPS, CIBERSAM, Barcelona, Spain; 5 Department of Psychosis Studies, Institute of Psychiatry, Psychology and Neuroscience, King’s College London, London, UK

**Keywords:** Mental Health, Psychiatry

## Abstract

The rapidly expanding field of mobile health (mHealth) seeks to harness increasingly affordable and ubiquitous mobile digital technologies including smartphones, tablets, apps and wearable devices to enhance clinical care. Accumulating evidence suggests that mHealth interventions are increasingly being adopted and valued by people living with serious mental illnesses such as schizophrenia and bipolar disorder, as a means of better understanding and managing their condition. We draw on experiences from three geographically and methodologically distinct mHealth studies to provide a pragmatic overview of the key challenges and considerations relating to the process of developing digital interventions for this population.

## The promise and challenges of mobile health (mHealth) in psychiatry

The opportunities presented by digital technologies including smartphones, apps and wearable devices for delivering new paradigms of care in people experiencing serious mental illness (SMI) have stimulated a surge of interest.^1^

The portable, connected nature of such devices enables the longitudinal, remote and high-resolution capture of clinical variables in ecologically valid settings—both *actively*, for example, via self-rated assessments, and *passively*, using sensors to sample objective markers of social, emotional and cognitive states, with low user burden. Information can be fed back to the patient and their clinical teams in real time, offering the opportunity to facilitate self-management, and trigger timely, preventative interventions. Mobile platforms may promote communication between patients and clinicians, and allow the delivery of therapies tailored to each user’s clinical status. The increasing ubiquity, affordability and ownership of digital technologies,^2^ including in populations with SMI^3–5^ has the potential to address the disparities in healthcare provision in underserved populations with SMI globally, including members of ethnic minorities, low-income groups, and individuals living in rural and low-resource settings.^6^ Several lines of evidence now indicate that people experiencing SMI already use,^3 5^ or are interested in using,^7^ mobile devices and web-based technology to manage their conditions, and that these are acceptable across a range of age, sex, educational level and clinical characteristics.^8^

As the field proliferates and establishes itself as a discipline in its own right, unique challenges emerge.^9 10^ A rush to implementation, with insufficient attention to design and usability during development, may be detrimental to the longer-term adoption of an intervention by patients and clinicians. Most interventions have been tested in small studies that report short-term feasibility and acceptability, largely with positive findings,^11 12^ raising questions around their performance over longer durations, in more heterogeneous patient groups. Evidence gathered through rigorous evaluation frameworks^13–15^ will be required to ensure purported benefits are realised, and that these outweigh potential harms such as breaches of security and privacy,^16^ devices and data that are of inadequate quality for the clinical application, and erosion of the therapeutic relationship.^17^

In this context, how do we optimise the process of designing effective digital solutions in a timely, cost-effective manner? Which clinical dimensions do we target? How do we create interventions that adapt to a shifting technological landscape, while retaining their core functionality? And how, when and to what extent do we integrate end users in the design process? Here, we explore these key considerations by drawing on the experiences gained from three methodologically and geographically diverse mHealth studies in people experiencing SMI (table 1), and offer a pragmatic and critical overview relating to the *process* of designing digital interventions for this population.

**Table 1:** Summary of characteristics of the three mHealth studies

	Sleepsight	SIMPLe	CLIMB
Disorder	Schizophrenia.	Bipolar disorder I and II.	Chronic psychotic disorders: schizophrenia, schizoaffective disorder, bipolar disorder with psychosis.
Target clinical dimension(s)	Rest-activity patterns, and their relation to symptomatic deterioration and relapse.	Self-reported positive and negative affect; relapse.	Social cognition and functioning.
Technologies	(1) Consumer wearable device with accelerometer and heart rate sensor; (2) Android smartphone provided by research team; (3) Custom designed smartphone application with self-rated symptom severity items, which also accessed smartphone sensors including accelerometer and smartphone usage meta-data.	(1) Participant’s own Android smartphone; (2) Custom designed smartphone application with self-rated symptom severity items, and tailored psychoeducational messages.	(1) iPad provided by research team; (2) Commercially available computerised social cognition training app; (3) Commercially available videoconferencing and social networking app.
Approach to development	Iterative focus groups informing app design and selection of mobile devices.	User-centred design, with iterative user involvement over the course of the study using online surveys, individual interviews and focus groups (figure 1).	Theory-driven intervention design, integrating structured training of social cognitive abilities with weekly group teletherapy with group texting. Online surveys that assess current social difficulties and patient-centred goals are administered to customise the group teletherapy sessions.
Project partners	**Academic:** Clinicians and bioinformaticians from King’s College London; app developers from Northwestern University, USA. **Non-academic:** Data access agreement with Fitbit.	**Academic:** Clinicians from the University of Barcelona, Spain, and collaborating academic centre in Santiago, Chile. **Non-academic:** App development was outsourced to a commercial entity in Paraguay.	**Academic:** Clinicians and data scientists at University of California at San Francisco. **Non-academic:** Posit science; Business associate agreement with Google.

## Defining the intervention

As the field gains legitimacy and attracts funding, there is a risk that technology is deployed for the sake of implementing technology alone, rather than being applied to target specific clinical problems. Clinician-researchers are however in a unique position to guide development of interventions by learning from patients, identifying areas of unmet clinical need, and then considering which of the various properties of mobile technologies might address these. The Sleepsight study (NM, London, UK, in submission) grew from the observation that sleep and circadian rhythm disturbance is a common but often neglected clinical complaint accompanying relapse in schizophrenia, and used the remote, passive, high-frequency, real time qualities of mobile and consumer wearable technologies to capture this dimension. The SIMPLe study^18 19^(DH-M, Barcelona, Spain), responded to the need to widen the reach and cost-effectiveness of psychoeducation in bipolar disorder, by building a bidirectional smartphone platform for gathering user-rated symptoms, and delivering personalised preventative psychoeducation in real time. The CLIMB study 20 (BB, San Francisco, USA) identified a need for new treatments to remediate impairments in social cognition and social functioning in psychosis-spectrum disorders—an underaddressed clinical dimension in this population—by harnessing properties inherent to digital technologies: remote interactive video communication, real time group messaging, and engaging cognitive training exercises delivered via a tablet device.

At this early stage, we suggest that a specific clinical dimension or unmet need should drive the research question; once the parameters and key variables are delineated, the development of a digital solution that addresses the area of need can begin. As the field progresses, individual tools can be amalgamated into multidimensional, modular and more flexible platforms.

## Developing partnerships

Establishing interdisciplinary collaborations that harness the expertise of clinicians, patients, data scientists, software engineers and user experience designers are critical in ensuring that an intervention meets scientific and technical standards. For example, the implementation of CLIMB (San Francisco, USA) relied on (1) an academic infrastructure that supported the incubation, development and evaluation of digital health technologies in clinical settings; (2) a long-standing collaboration between the academic research group and Posit Science, a neurotechnology company that provided customised software free of charge; (3) a business associate agreement with Google, which enabled the free use and integration of secure peer-to-peer communication technology; and (4) the geographical proximity of these entities, which accelerated development and deployment of the intervention.

However, traditions of scientific independence, difficulties in sharing implicit knowledge and organisational barriers can form obstacles to collaboration. Employing ‘team science’ principles^21^—scheduling meetings and initiatives to promote interdisciplinary dialogue, and ultimately the formation of a collective knowledge base—can help to align objectives and nurture effective collaborations. Before any work begins, it is essential that agreement is reached between collaborators on issues of payment, academic credit, authorship, intellectual property, and data ownership, storage and security. Another critical issue which can be detrimental for a project is if a partner withdraws support, as was the case in the SIMPLe project. Where possible, signing a partnership agreement may mitigate this risk, together with contingency planning in case of this eventuality.

Although CLIMB benefited from the unusually technology-rich environment of San Francisco, digital interventions can be developed in tech hubs and rapidly disseminated worldwide, including to low-income and middle-income countries^6^ and areas of conflict or disaster.^22^ Effective collaborations can also be formed internationally: in addition to partners in the main academic institution in London, the Sleepsight team benefited from working with developers from Northwestern University, Chicago, USA with expertise in mHealth app development. While we realise that not all digital health projects require this infrastructure to be successful, we believe that fostering trusted, multidisciplinary networks greatly enhances the development process.

## Involving patients

mHealth interventions are likely to be adopted for long-term use only if they provide intrinsic value for the user in managing their condition, or improve critical aspects of their functioning and well-being. Involving end-users in the co-development of digital interventions has therefore become axiomatic to this goal, however, it is not yet clear how this is best implemented, and only a limited number of studies to date have reported user-involvement.^23^

**Figure 1:**
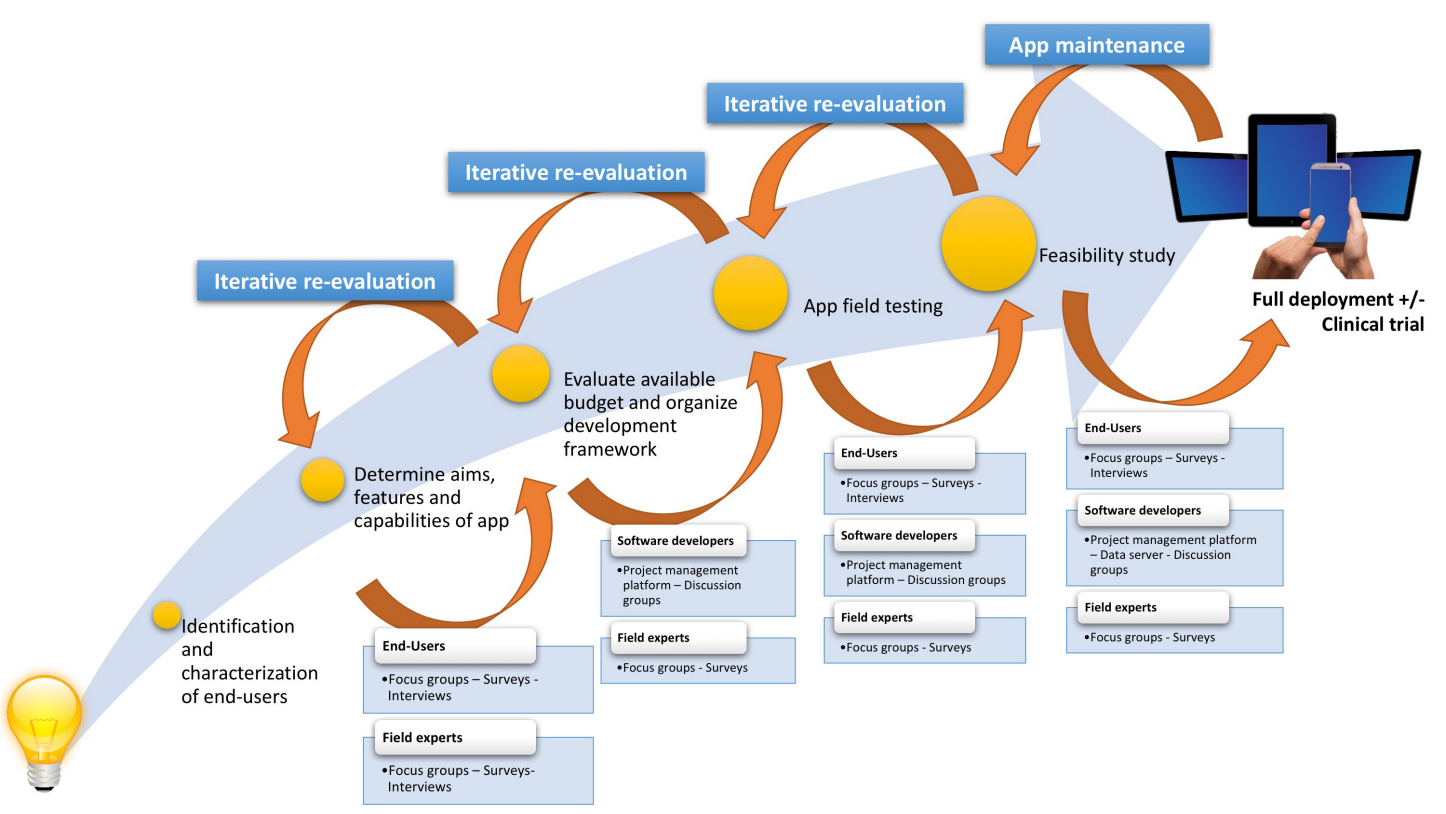
User-centred development pathway for the SIMPLe study.

Truly user-centred design (UCD) involves a bottom-up approach, where users are integral to forming the research agenda, clinical focus and technology selection over the entirety of the development cycle. From the project’s infancy, SIMPLe employed online surveys, focus groups and individual interviews in an iterative fashion, to gather opinion, implement prototypes, then evaluate, refine and re-evaluate these (figure 1). A setback in the process was not considered a failure, but an opportunity to optimise long-term acceptability. UCD is likely to have contributed to the high adherence rate of 74% over the 3-month study, however it was intensive of the project’s time, and financial and human resources,^19^ and added significant complexity to the process. Several features were developed and tested, but discarded from the final version of the app.

Sleepsight and CLIMB also found encouraging adherence rates of over 80%, but involved a more top-down approach, where patients and public were consulted through user groups on aspects of the design once an overarching approach had been formulated by the research team. This suggests that a more conservative approach to patient involvement also resulted in acceptable interventions, and that the optimal balance between bottom-up and top-down approaches remains unclear. In both cases, user groups tended to be composed of motivated ‘patient-champions’ who may not be representative of the wider patient population.

## Native or existing software platforms?

Do we build native software platforms (SIMPLe and Sleepsight), or leverage existing technology and repurpose it to meet the needs of the project (CLIMB)?

While building native software requires up-front expenditures of time, energy and capital, it also allows for specific difficulties of people with SMI to be taken into account, including cognitive impairments, poor technology literacy and text reading level.^24^ This is a critical aspect, as the potential of digital interventions is known to be mitigated by design and usability issues: incorporating the principles of navigational simplicity, text reading ease and comprehensibility into the development of native technology for SMI was shown to increase the acceptability of the intervention.^24^ There are, nonetheless, potential risks associated with building native technology for SMI. For example, technological advances may make the prototype for the intervention already obsolete and uncompetitive by the time the development process is complete, with obvious implications on the acceptability and scalability of the intervention. One strategy for overcoming this limitation is to outsource design and development to established firms, so that the intervention is regularly maintained and enhanced, however this usually requires additional costs that may be unaffordable to early stage researchers.

An alternative is to capitalise on existing solutions that are in general use, provided they meet the specific needs and goals of the study, are secure, and compliant with ethical and clinical governance structures. Advantages include a significant reduction in costs and the possibility to promptly test the feasibility and acceptability of the technology in the clinical population. However, researchers have very little control over the development process, and may need to adapt the intervention to the rapidly changing features of the digital tool, in turn creating problems with consistency of study procedures and replicability of findings.

In both scenarios, allowing interoperability between mobile operating systems is desirable, particularly android and iOS systems, in order to maximise the reach and generalisability of the intervention. Both CLIMB and SIMPLe employed software that could be freely downloaded to any smartphone, and other studies have used cost-effective approaches such as text message rating of mood,^25^ the utility of which has also been demonstrated in low-income and middle-income settings.^26^

## Consumer or clinical grade devices?

A key objective for the field is developing interventions which are affordable and acceptable for extended use. For studies such as Sleepsight, which capture passive behavioural and physiological variables from accelerometer, light and GPS sensors, validated ‘clinical grade’ wearable and smartphone devices which meet these criteria are not currently available. Similarly, developing bespoke devices is a prohibitively lengthy and expensive process. Sleepsight, like several related studies,^27 28^ therefore harnessed lower-cost consumer technologies, which capitalises on the functionality, user experience research that has been invested in their development. However, these devices are not validated in clinical populations, and in the case of wearable devices, data are preprocessed on the device using unpublished algorithms, to which the research team do not have access. This raises questions around their validation, data quality, replicability and scientific acceptability.

We suggest that consumer devices have a useful role, as long as we are conscious of their limitations. First, the technology needs to be ‘good enough’ to address the clinical question. For Sleepsight, consumer wearable devices are likely to be adequate for estimation of gross rest-activity patterns, and detecting variability in this signal against background noise. However, they are unable to provide raw accelerometer data that permit reliable estimation of sleep, analysis of variability in motor activity on shorter timescales, or beat-to-beat variability in heart rate. Second, we attempted to minimise variability between devices by providing all participants with standardised study devices, and by primarily examining longitudinal within-person variation. Third, given the rapid development cycle of consumer devices, the platform was designed to integrate with new devices as they emerge, thus ‘future-proofing’ the intervention.

As the technological landscape rapidly evolves, new opportunities will arise for delivering digital tools for mental health. Closer collaboration between academic and industry partners will be necessary in fusing the advantages of consumer and clinical grade technologies in the development of devices that integrate seamlessly into the user’s everyday life, while delivering clinical-quality data.

## Conclusions

In attending to the many challenges facing the field—ensuring privacy and security,^16^ understanding new types of data,^29^ integrating mHealth into existing clinical infrastructure and transitioning from studies of feasibility to those of effectiveness—the importance of the underlying process of developing digital tools should not be overlooked. Each point discussed above relates ultimately to the production of digital interventions that are valued by patients and clinicians, and therefore compatible with long-term use, while meeting clinical and scientific standards.

The successful development of a digital intervention for people experiencing SMI requires the coordinated activity of clinical researchers with patients, clinicians and the technology sector. The intervention should emerge in response to a symptom dimension or unmet clinical need, which resonates with the patient group it is intended to serve. The native development of a device or software-based intervention is an expensive and laborious process that can limit the quality, life span and breadth of the product, with important implications on its acceptability and scalability. Therefore, building strategic and appropriately formalised academic-industry partnerships is a viable alternative that can result in customised digital tools that are more easily adopted and disseminated.

Finally, the flow of knowledge and technology between stakeholders in academia and industry, for example, by sharing code via open-source frameworks, will be critical to success. Adapting, testing and improving interventions in different settings globally will enhance transparency, replicability, efficiency and pace of development for this exciting field.
